# mHealth App Barriers, Usability, and Personalization: A Cross-Sectional Study from Egypt and Saudi Arabia

**DOI:** 10.3390/jpm12122038

**Published:** 2022-12-09

**Authors:** Ahmed Arafa, Zahraa M. Mostafa, Haytham A. Sheerah, Fahad Alzahrani, Yasir Almuzaini, Shaimaa Senosy, Radwa Ibrahim Ali Hassan

**Affiliations:** 1 Department of Preventive Cardiology, National Cerebral and Cardiovascular Center, Suita 564-8565, Japan; 2 Public Health, Department of Social Medicine, Osaka University Graduate School of Medicine, Suita 565-0871, Japan; 3 Department of Public Health and Community Medicine, Faculty of Medicine, Beni-Suef University, Beni-Suef 2722165, Egypt; 4 International Collaborations Ministry of Health, Riyadh 11176, Saudi Arabia; 5 Global Center for Mass Gatherings Medicine Ministry of Health, Riyadh 11176, Saudi Arabia; 6 Ministry of Health, Riyadh 11176, Saudi Arabia; 7 Department of Public Health and Community Medicine, Faculty of Medicine, Cairo University, Cairo 12613, Egypt

**Keywords:** mHealth, eHealth, barriers, usability, personalization

## Abstract

Mobile health (mHealth) has emerged as a substantial segment of eHealth. Herein, we conducted a cross-sectional study to investigate mHealth app barriers, usability, and personalization in Egypt and Saudi Arabia. We used a Google survey to recruit participants from both countries between the 15th of September and the 15th of October 2022. Among 299 participants (247 from Egypt and 52 from Saudi Arabia), aged ≥ 18 years, 27.4% reported mHealth app use. In the age-, sex-, and country-adjusted regression models, age > 25 years: OR (95% CI) = 1.98 (1.11, 3.54), residing in Saudi Arabia: OR (95% CI) = 4.33 (2.22, 8.48), and physical activity: OR (95% CI) = 2.53 (1.44, 4.44) were associated with mHealth app use. The main mHealth app purposes were lifestyle promotion (35.4%), diet and nutrition (30.5%), and administrative services (13.4%). On a scale from 20 to 100, mHealth app usability scores were 46.3% (20–40), 7.3% (41–60), 31.7% (61–80), and 14.7% (81–100). According to 93.9% of users, mHealth app features were modifiable to meet personal health goals, while 37% stated that mHealth apps helped them set new personal health goals. In conclusion, age, residing in Saudi Arabia (compared to Egypt), and physical activity were positively associated with mHealth app use. mHealth app feature personalization and helping users set new personal health goals were largely reported, suggesting that mHealth has the potential to help put personalized healthcare into practice.

## 1. Introduction

Per the World Health Organization’s Global Observatory for eHealth, mobile health (mHealth) is defined as medical and public health practices supported by mobile devices [1]. Alongside the extending internet coverage and increasing smartphone ownership in developed and developing countries, mHealth has emerged as a significant element of eHealth [1,2]. The COVID-19 pandemic has highlighted the mHealth role and made it more integrated into healthcare [3,4,5]. 

mHealth poses several well-documented advantages such as minimizing the spread of infection, saving time, convenience, and cost-effectiveness, yet its usability remains controversial, especially among people who are not familiar with mobile apps [6,7,8]. On the other hand, the medical practice has been shifting from a traditional approach based on population-derived guidelines to a personalized paradigm providing tailored preventive and therapeutic strategies according to the individual’s lifestyle, medical condition, and genetic profile [9]. mHealth is suggested to enhance this shift [10]. Still, involving mHealth in healthcare systems faces many sociocultural, medicolegal, ethical, technical, and financial barriers, especially in developing countries [6,7,8].

Egypt and Saudi Arabia have been witnessing a digital transformation in the healthcare section with increasing reliance on mHealth apps to improve healthcare access, patient communication and monitoring, treatment adherence, and health education [11,12,13,14,15,16,17]. However, a few studies investigated mHealth app use in both countries [11,12,18,19,20]. Most of these studies assessed eHealth or telemedicine in general rather than mHealth, did not use validated tools to measure usability, and did not investigate whether eHealth forms could serve the transformation into personalized healthcare. Therefore, we conducted this study to investigate factors associated with mHealth app use in Egypt and Saudi Arabia and assess its usability and personalization.

## 2. Methods

### 2.1. Participants

In this cross-sectional study, we included people residing in Egypt or Saudi Arabia who were aged ≥ 18 years old. First, we created a Google survey and uploaded the survey link to several social network groups hosting Egyptian and Saudi people. Social network use is widespread in both countries [21,22]. Then, we asked eligible people to fill out the online survey and send the survey link to their friends and relatives. The responses were collected between the 15th of September and the 15th of October 2022. 

We calculated the sample size using the Epi-Info version 7 StatCalc, which is available from the Centers for Disease Control (CDC) and the WHO. The prevalence of mHealth app use is not precisely known in both countries. However, a recent study estimated that 23.8% of healthcare workers in Egypt were using mHealth during the COVID-19 pandemic [12]. Eventually, we applied the following criteria for sample size calculation: confidence level (95%), a margin of error (5%), and mHealth app use prevalence (25%). The minimum number of necessary samples to meet the desired statistical constraints was 289.

### 2.2. Data Collection

We designed a questionnaire composed of 3 sections for data collection. 

#### 2.2.1. Section I

It included a detailed explanation of the study objectives, steps, and eligibility criteria. Participants who approved the study conditions were transferred to the next section(s). 

#### 2.2.2. Section II

It included questions about the sociodemographic characteristics of participants including age (years), sex, country (Egypt or Saudi Arabia), residence (urban or rural), social status (single/others or married), job (student [medical field or non-medical field], worker [medical field or non-medical field], or house maker), income (sufficient, hardly sufficient, or insufficient), smoking behavior (never, former, or current), physical activity (yes or no), having a chronic disease (yes [and mention] or no), internet use (hours/day), and mHealth app use (yes or no). Participants who reported mHealth app use were transferred to the following section. 

#### 2.2.3. Section III

Since many mHealth app users were using >1 mHealth app, section III included questions related to the most used mHealth app only. We divided this section into 3 parts: section III-1 (purposes): It assessed the purposes of mHealth apps (administrative services such as medical reservations and vaccination appointments and certificates; lifestyle promotion not including nutrition and diet; healthy nutrition and die; or others [and mention]); Section III-2 (usability): It included the mHealth App Usability Questionnaire (MAUQ), which consisted of 5 items assessing ease of use, 7 items assessing interface and satisfaction, and 6 items assessing usefulness. Participants had to decide how much each item applied to them on a Likert scale from 1 to 5, where 1 referred to “strongly disagree”, 2 “agree”, 3 “neutral”, 4 “agree”, and 5 “strongly agree”. The scores were translated to a scale from 20 to 100, and higher scores indicated higher usability. The Cronbach alpha value of the entire questionnaire per the original questionnaire was 0.914 [23] and the corresponding value in the current study was even higher, indicating a strong internal consistency; and Section III-3 (personalization): It included a question assessing participants’ opinions on whether the features of mHealth apps could be modified to meet personal health goals (yes or no) and a question assessing whether mHealth apps could help them set new personal health goals (strongly disagree, disagree, neutral, agree, and strongly agree). 

We programmed the Google survey to make all questions mandatory. Before distributing the questionnaire, the research team checked its face and content validity while the questionnaire understandability was examined on a pilot of 10 participants. At the end of the survey period, 317 responses were received, yet we excluded 18 responses for not meeting our eligibility criteria, leaving 299 responses for analysis.

### 2.3. Statistical Analyses

We used logistic regression analyses to calculate the odds ratios (ORs) with 95% confidence intervals (CIs) of different sociodemographic factors for mHealth app use. The results were adjusted for age, sex, and country. We displayed the usability scores (20–40, 41–60, 61–80, and 81–100) and the response to the statement of setting new personal health goals using bar charts, purposes and feature personalization using pie charts, and the association between usability scores and setting new personal health goals using box plots. The *t*-test was applied to compute the usability scores by sociodemographic characteristics and the one-way ANOVA with the Bonferroni post hoc correction to compare the usability scores across different responses to the statement of setting personal health goals. We used the Statistical Package for Social Science (SPSS) released in 2013 (IBM SPSS Statistics for Windows, Version 22.0, IBM Corporation, Armonk, NY, USA) for data analysis.

## 3. Results

Among the 299 participants (aged 29.8 ± 11.6 years), 59.9% were women, 82.6% were residing in Egypt (17.4% in Saudi Arabia), 42.1% were university students, and 27.4% reported mHealth app use (Table 1).

In the unadjusted regression model, age > 25 years: OR (95% CI) = 2.77 (1.61, 4.76), residing in Saudi Arabia: 5.67 (3.01, 10.69), living in an urban area: 3.08 (1.57, 6.04), being a worker or house maker: 1.83 (1.10, 3.06), having sufficient income: 2.96 (1.67, 5.24), and practicing physical activity: 2.23 (1.32, 3.76) were associated with higher odds of mHealth app use. After adjustment for age, sex, and country, the positive associations remained significant only in age > 25 years: 1.98 (1.11, 3.54), residing in Saudi Arabia: 4.33 (2.22, 8.48), and physical activity: 2.53 (1.44, 4.44) (Table 2).

**Figure 1:**
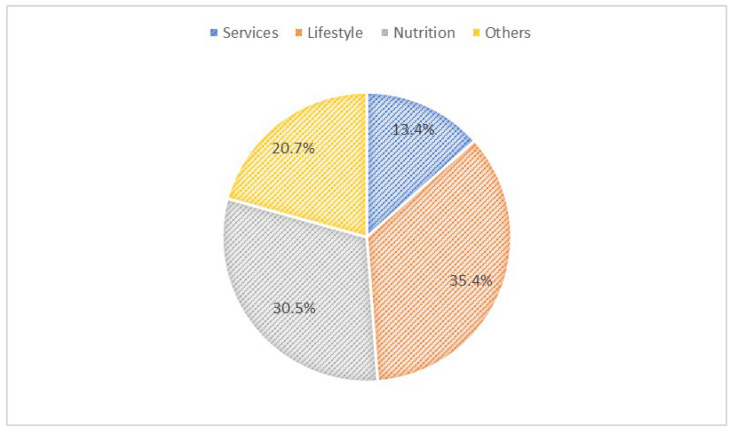
Main purposes of mHealth apps.

**Figure 2:**
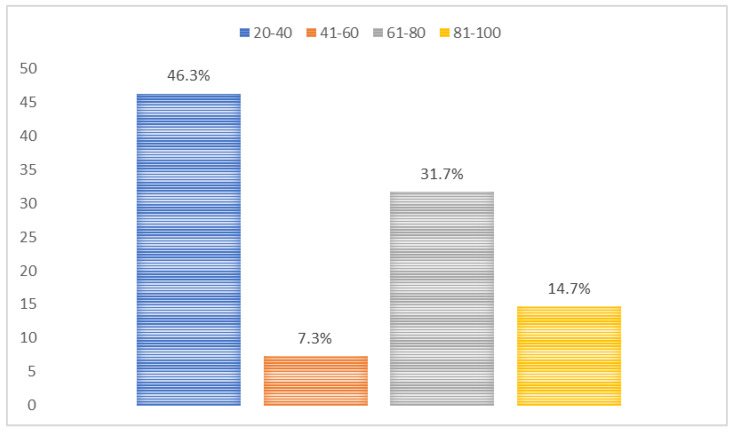
Usability of mHealth apps on a scale from 20 to 100.

**Figure 3:**
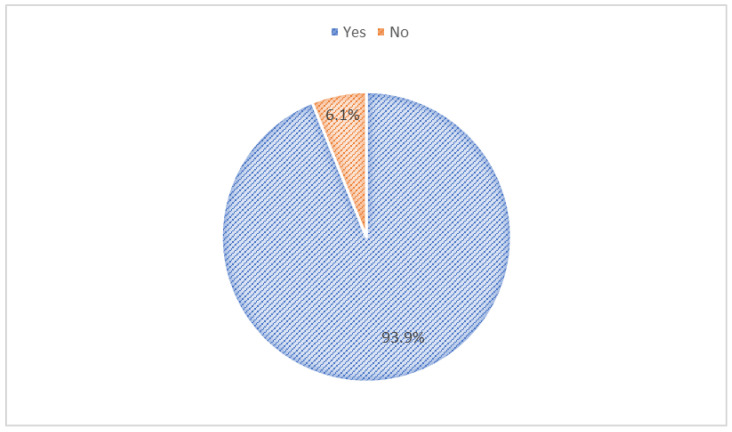
The ability to modify the features of mHealth apps to meet personal health goals.

**Figure 4:**
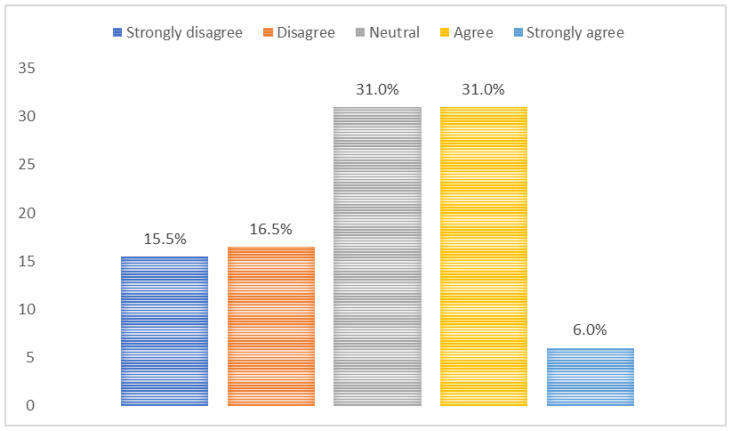
The ability of mHealth apps to help users set personal health goals that they were not able to do.

**Figure 5:**
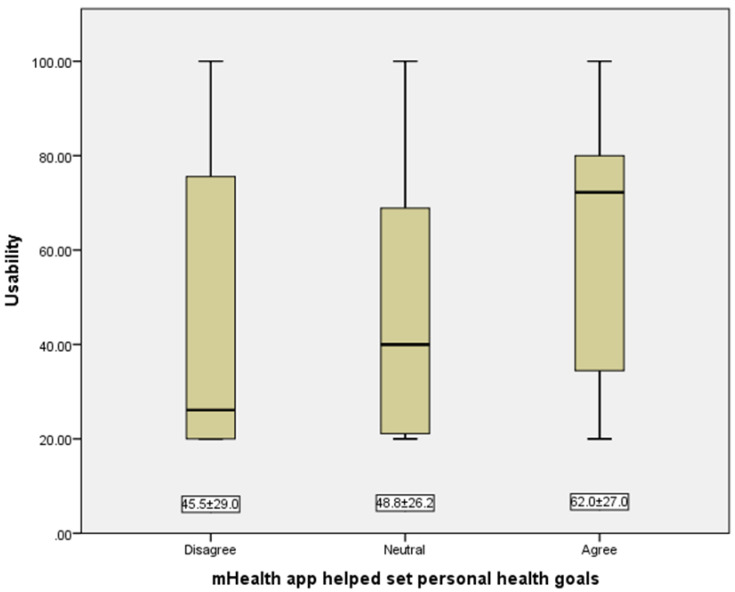
The association between usability scores and setting personal health goals among mHealth app users. Using one-way ANOVA with the Bonferroni post hoc correction, the *p*-values were as follows: disagree vs. neutral = 0.999, neutral vs. agree = 0.230, and disagree vs. agree = 0.081).

Among mHealth app users (*n* = 82), the main purposes of mHealth apps were administrative services (13.4%), lifestyle promotion (35.4%), nutrition and diet (30.5%), and others (20.7%) (Figure 1). On a scale from 20 to 100, the usability scores of mHealth apps were 46.3% (20–40), 7.3% (41–60), 31.7% (61–80), and 14.7% (81–100) (Figure 2). mHealth app users who reported sufficient income, smoking, and having chronic diseases had higher usability scores (*p*-values < 0.05) (Table 3). Most mHealth app users (93.9%) reported that their features could be modified to meet personal health goals (Figure 3). More than a third of participants (37.0%) agreed with the statement suggesting that mHealth apps helped them set new personal health goals while 32.0% disagreed with this statement (Figure 4). The usability scores (mean ± Sd) varied across different responses to the previous statement: disagree 45.5 ± 29.0, neutral: 48.8 ± 26.2, and agree: 62.0 ± 27.0 (*p*-values: disagree vs. neutral = 0.999, neutral vs. agree = 0.230, and disagree vs. agree = 0.081) (Figure 5). 

## 4. Discussion

The growing field of mHealth with its technological advancements could help improve healthcare and promote a healthy lifestyle. This study investigated barriers to mHealth app use in a nonrandom cohort from Egypt and Saudi Arabia and described mHealth app usability and personalization. 

Our results indicated that participants who were >25 years, residing in Saudi Arabia (compared to Egypt), and practicing physical activity were more likely to use mHealth apps. The positive association between age and mHealth app use could reflect the increasing need for lifestyle modification with age. However, it could be explained by the fact that many mHealth apps are not completely free and younger participants who were typically university students did not have enough money to pay for these apps or upgrade their features. Besides, the higher mHealth app use among participants residing in Saudi Arabia may indicate the rapid digitalization of the country’s healthcare system [16,17,18]. In line with our results, the association between mHealth apps and promoting physical activity was heavily described in the literature [24]. A meta-analysis of 118 randomized controlled trials showed that mHealth interventions could foster increases in physical activity, and their effects could be maintained for a long time [25].

However, less than half of the mHealth app users in our study reported adequate usability scores. This finding highlights the need for developing easier to use mHealth apps in Egypt and Saudi Arabia. In a recent study including 318 participants from Saudi Arabia, inability to recover from mistakes, inconsistency in navigation, and lack of all necessary functions were the main complaints while using mHealth apps [20]. We could also detect a positive association between sufficient income and higher usability scores that could be explained by the possibly better education among participants with sufficient income, which made it easier for them to use mHealth apps. In addition, richer participants might have been more able to buy smartphones with better features and pay for mHealth apps with higher usability. 

While most participants reported that the features of mHealth apps were modifiable to meet personal health goals, only a third of them stated that mHealth apps helped them set personal health goals. However, these findings give the impression that participants believed mHealth apps could help them personalize health goals. We also noted that setting new personal health goals using mHealth apps was positively associated, however, statistically insignificant, with usability scores. This means that participants who found mHealth apps easier to use were able to make the best use of mHealth apps in terms of setting new personal health goals. Therefore, the possible link between mHealth usability and personalization should be considered while updating or developing mHealth apps. Of note, the role of mHealth apps in the transformation from traditional to personalized medicine was barely described [10,26]; therefore, more research is needed to understand the perceptions of healthcare providers and patients and investigate the technical, legislative, ethical, and sociocultural factors related to this role.

Although our study investigated many factors related to mHealth app use among underrepresented populations, several limitations should be addressed. First, we used a non-probability snowball sampling approach to recruit participants, which undermined the representativeness of our study population. It could be speculated that mHealth app users were more motivated to participate in this study. Additionally, the online data collection could be accompanied by non-response bias as non-respondents might have carried different characteristics than respondents [27]. To minimize this bias, we did not ask participants to unveil their identities. We also forwarded the survey link via different social networks to access Egyptian and Saudi people from different social backgrounds. Still, it was obvious that our study population had a higher educational level and better lifestyle behaviors (low smoking and high physical activity prevalence) than the overall population in both countries. Second, because of the limited number of mHealth app users, we were not able to perform multivariable-adjusted regressions investigating associations with mHealth app usability scores, so we performed *t*-tests instead. For the same reason, we could not study factors related to feature personalization or setting new personal health goals and provided simple descriptions instead. Third, since many mHealth app users were using > 1 mHealth app, we asked them only to report the usability and personalization of the most used mHealth app. mHealth apps may differ in terms of purposes and usability, suggesting bias. Fourth, due to the cross-sectional design of this study, we could not assign a temporal association between mHealth app use and physical activity. On the one hand, mHealth apps might have encouraged users to engage in physical activity. On the other hand, physically active people might have used mHealth apps to promote their practice. Fifth, the observational nature of this study could hide confounders. 

In conclusion, age, residing in Saudi Arabia (compared to Egypt), and physical activity were positively associated with mHealth app use. Although the reported usability scores were not high, mHealth apps showed a recognizable potential for feature personalization and helping users set new personal health goals. Still, larger studies with prospective designs are needed to confirm our findings and investigate in-depth factors related to mHealth app personalization. Since many sociocultural factors related to mHealth use barriers and personalization are difficult to be assessed using simple questionnaires or scales, especially in conservative societies, future qualitative studies are warranted. It was suggested that improving the telemedicine infrastructure by optimizing the medical datasets and improving remote medicine dataset transmission and processing may facilitate mHealth use [28]. We believe that mHealth apps could improve healthcare delivery in Egypt and Saudi Arabia; however, barriers to mHealth app use and factors hindering its usability should be managed before applying mHealth on a widescale.

## Figures and Tables

**Table 1:** Sociodemographic characteristics of participants (*n* = 299).

	Characteristics	Frequency (%)
Age (years)	18–25	144 (48.2)
	>25	155 (51.8)
Sex	Women	179 (59.9)
	Men	120 (40.1)
Country	Egypt	247 (82.6)
	Saudi Arabia	52 (17.4)
Residence	Rural	87 (29.1)
	Urban	212 (70.9)
Social status	Single	171 (49.2)
	Married	152 (50.8)
Education	University or higher	295 (98.7)
	Elementary	4 (1.3)
Job	Student	126 (42.1)
	Worker/House maker	173 (57.9)
Income	Hardly sufficient/Insufficient	152 (50.8)
	Sufficient	147 (49.2)
Smoking	No	277 (92.6)
	Yes	22 (7.4)
Physical activity	No	192 (64.2)
	Yes	107 (35.8)
Chronic diseases	No	226 (75.6)
	Yes	73 (24.4)
Internet use (h/day)	1–3	67 (22.4)
	>3	232 (77.6)
mHealth app use	No	217 (72.6)
	Yes	82 (27.4)

Mean ± Sd (range) of age = 29.8 ± 11.6 (18–72) years and internet use = 5.8 ± 2.9 (1–12) h/day.

**Table 2:** Factors associated with mHealth app use.

Factors		Users%	Non-Users%	Model I OR (95% CI)	Model II OR (95% CI)
Age (years) *	18–25	30.5	54.8	Ref	Ref
	>25	69.5	45.2	**2.77 (1.61, 4.76)**	**1.98 (1.11, 3.54)**
Sex	Women	53.7	62.2	Ref	Ref
	Men	46.3	37.8	1.42 (0.85, 2.38)	1.22 (0.70, 2.12)
Country	Egypt	62.2	90.3	Ref	Ref
	Saudi Arabia	37.8	9.7	**5.67 (3.01, 10.69)**	**4.33 (2.22, 8.48)**
Residence	Rural	14.6	34.6	Ref	Ref
	Urban	85.5	65.4	**3.08 (1.57, 6.04)**	1.71 (0.80, 3.64)
Social status	Single	46.3	61.3	Ref	Ref
	Married	53.7	38.7	**1.83 (1.10, 3.06)**	1.01 (0.48, 2.15)
Job	Student	24.4	48.8	Ref	Ref
	Worker/House maker	75.6	51.2	**2.96 (1.67, 5.24)**	1.55 (0.59, 4.10)
Income	Hardly sufficient/Insufficient	36.6	56.2	Ref	Ref
	Sufficient	63.4	43.8	**2.23 (1.32, 3.76)**	1.63 (0.93, 2.86)
Smoking	No	78.8	94.5	Ref	Ref
	Yes	12.2	5.5	2.37 (0.98, 5.73)	1.28 (0.45, 3.65)
Physical activity	No	48.8	70.0	Ref	Ref
	Yes	51.2	30.0	**2.46 (1.46, 4.14)**	**2.53 (1.44, 4.44)**
Chronic diseases	No	73.2	76.5	Ref	Ref
	Yes	26.8	23.5	1.19 (0.67, 2.13)	1.06 (0.56, 1.99)
Internet use (hours/day) **	1–3	19.5	23.5	Ref	Ref
	> 3	80.5	76.5	1.27 (0.68, 2.38)	1.35 (0.68, 2.71)

Model I: Unadjusted. Model II: Adjusted for age, sex, and country. * OR (95% CI) per 1-year increase in age: 1.032 (1.010, 1.054) in the unadjusted model and 1.019 (0.995, 1.043) in the model adjusted for sex and country. ** OR (95% CI) per 1-h/day increase in internet use: 1.079 (0.991, 1.175) in the unadjusted model and 1.058 (0.959, 1.168) in the model adjusted for, age, sex, and country. Bold: Statistically significant.

**Table 3:** Factors associated with mHealth app usability among mHealth app users (*n* = 82).

Factors		Usability Mean ± Sd	*p*-Value
Age (years)	18–25	51.5 ± 29.1	0.812
	>25	53.1 ± 27.8	
Sex	Women	48.2 ± 26.7	0.126
	Men	57.7 ± 29.0	
Country	Egypt	49.0 ± 27.3	0.133
	Saudi Arabia	58.6 ± 28.6	
Residence	Rural	38.4 ± 20.0	0.058
	Urban	55.0 ± 28.6	
Social status	Single	53.5 ± 29.0	0.784
	Married	51.8 ± 27.5	
Job	Student	55.8 ± 30.1	0.561
	Worker/House maker	51.6 ± 27.5	
Income	Hardly sufficient/Insufficient	39.7 ± 24.8	**0.001**
	Sufficient	60.0 ± 27.3	
Smoking	No	50.2 ± 27.4	**0.035**
	Yes	70.0 ± 27.4	
Physical activity	No	57.3 ± 28.4	0.135
	Yes	48.1 ± 27.2	
Chronic diseases	No	48.6 ± 27.5	**0.030**
	Yes	63.6 ± 27.0	
Internet use (h/day)	1–3	43.3 ± 28.2	0.139
	> 3	54.8 ± 27.7	

Bold: Statistically significant.

## Data Availability

Available upon a reasonable request.

## References

[1] Seewon R. (2012). Book review: mHealth: New horizons for health through mobile technologies: Based on the findings of the Second Global Survey on eHealth (Global Observatory for eHealth Series, Volume 3). Healthc. Inform. Res..

[2] Marcolino M.S., Oliveira J.A.Q., D’Agostino M., Ribeiro A.L., Alkmim M.B.M., Novillo-Ortiz D. (2018). The impact of mhealth interventions: Systematic review of systematic reviews. JMIR Mhealth Uhealth.

[3] Taha A.R., Shehadeh M., Alshehhi A., Altamimi T., Housser E., Simsekler M., Alfalasi B., Al Memari S., Al Hosani F., Al Zaabi Y. (2022). The integration of mHealth technologies in telemedicine during the COVID-19 era: A cross-sectional study. PLoS ONE.

[4] Ibrahim A.E., Magdy M., Khalaf E.M., Mostafa A., Arafa A. (2021). Teledermatology in the time of COVID-19. Int. J. Clin. Pract..

[5] Iyengar K., Upadhyaya G.K., Vaishya R., Jain V. (2020). COVID-19 and applications of smartphone technology in the current pandemic. Diabetes Metab. Syndr..

[6] Koh J., Tng G.Y.Q., Hartanto A. (2022). Potential and pitfalls of mobile mental health apps in traditional treatment: An umbrella review. J. Pers. Med..

[7] Kruse C., Betancourt J., Ortiz S., Valdes Luna S.M., Bamrah I.K., Segovia N. (2019). Barriers to the use of mobile health in improving health outcomes in developing countries: Systematic review. J. Med. Internet Res..

[8] Nittari G., Khuman R., Baldoni S., Pallotta G., Battineni G., Sirignano A., Amenta F., Ricci G. (2020). Telemedicine practice: Review of the current ethical and legal challenges. Telemed. J. e-Health.

[9] Golubnitschaja O., Baban B., Boniolo G., Wang W., Bubnov R., Kapalla M., Krapfenbauer K., Mozaffari M.S., Costigliola V. (2016). Medicine in the early twenty-first century: Paradigm and anticipation—EPMA position paper 2016. EPMA J..

[10] Battineni G., Sagaro G.G., Chintalapudi N., Amenta F. (2021). The benefits of telemedicine in personalized prevention of cardiovascular diseases (CVD): A systematic review. J. Pers. Med..

[11] Alboraie M., Allam M.A., Youssef N., Abdalgaber M., El-Raey F., Abdeen N., Mahdy R.E., Elshaarawy O., Elgebaly A., Haydara T. (2021). Knowledge, applicability, and barriers of telemedicine in Egypt: A national survey. Int. J. Telemed. Appl..

[12] Alboraie M., Abdalgaber M., Youssef N., Moaz I., Abdeen N., Abosheaishaa H.M., Shokry M.T., El-Raey F., Asfour S.S., Abdeldayem W.A. (2022). Healthcare providers’ perspective about the use of telemedicine in Egypt: A national survey. Int. J. Telemed. Appl..

[13] Al-Hazmi A.M., Sheerah H.A., Arafa A. (2021). Perspectives on telemedicine during the era of COVID-19; what can Saudi Arabia do?. Int. J. Environ. Res. Public Health.

[14] Alhodaib H., Alanzi T.M. (2021). Understanding the impact of digital health strategies during the COVID-19 outbreak in Saudi Arabia. Risk Manag. Healthc. Policy.

[15] Al-Kahtani N., Alruwaie S., Al-Zahrani B.M., Abumadini R.A., Aljaafary A., Hariri B., Alissa K., Alakrawi Z., Alumran A. (2022). Digital health transformation in Saudi Arabia: A cross-sectional analysis using Healthcare Information and Management Systems Society’ digital health indicators. Digit. Health.

[16] Alghamdi S.M., Alsulayyim A.S., Alqahtani J.S., Aldhahir A.M. (2021). Digital health platforms in Saudi Arabia: Determinants from the COVID-19 pandemic experience. Healthcare.

[17] Alghamdi S.M., Alqahtani J., Aldhahir A.M. (2020). Current status of telehealth in Saudi Arabia during COVID-19. J. Fam. Community Med..

[18] Kaliyadan F., Al Ameer A.M., Al Ameer A., Al Alwan Q. (2020). Telemedicine practice in Saudi Arabia during the COVID-19 pandemic. Cureus.

[19] Alanezi F. (2021). Factors affecting the adoption of e-health system in the Kingdom of Saudi Arabia. Int. Health.

[20] Alanzi T.M. (2022). Users’ satisfaction levels about mHealth applications in post-Covid-19 times in Saudi Arabia. PLoS ONE.

[21] Arafa A., Saif S.A., Ramadan A., Rashed T., Ahmed S., Taha M. (2019). Problematic internet use: A cross-sectional study on a model from university students in Egypt. Int. J. Adolesc. Med. Health.

[22] AlMuammar S.A., Noorsaeed A.S., Alafif R.A., Kamal Y.F., Daghistani G.M. (2021). The use of internet and social media for health information and its consequences among the population in Saudi Arabia. Cureus.

[23] Zhou L., Bao J., Setiawan I.M.A., Saptono A., Parmanto B. (2019). The mHealth App Usability Questionnaire (MAUQ): Development and validation study. JMIR Mhealth Uhealth.

[24] Carter D.D., Robinson K., Forbes J., Hayes S. (2018). Experiences of mobile health in promoting physical activity: A qualitative systematic review and meta-ethnography. PLoS ONE.

[25] Mönninghoff A., Kramer J.N., Hess A.J., Ismailova K., Teepe G.W., Tudor Car L., Müller-Riemenschneider F., Kowatsch T. (2021). Long-term effectiveness of mHealth physical activity interventions: Systematic review and meta-analysis of randomized controlled trials. J. Med. Internet Res..

[26] Record J.D., Ziegelstein R.C., Christmas C., Rand C.S., Hanyok L.A. (2021). Delivering personalized care at a distance: How telemedicine can foster getting to know the patient as a person. J. Pers. Med..

[27] Arafa A.E., Anzengruber F., Mostafa A.M., Navarini A.A. (2019). Perspectives of online surveys in dermatology. J. Eur. Acad. Dermatol. Venereol..

[28] Ahmed S.T., Sandhya M., Sankar S. (2019). A Dynamic MooM Dataset Processing Under TelMED Protocol Design for QoS Improvisation of Telemedicine Environment. J. Med. Syst..

